# Draft genome sequences of clinical strains of *Brucella melitensis* BBM21 and BVC23 recovered from blood cultures

**DOI:** 10.1128/mra.00040-26

**Published:** 2026-05-18

**Authors:** Harshita Mogaveera, Venkat Abhiram Earny, Sudipta Patra, Chiranjay Mukhopadhyay, Manjunath Joshi, Vandana Kalwaje Eshwara

**Affiliations:** 1 Department of Microbiology, Kasturba Medical College, Manipal Academy of Higher Education 76793, Manipal, India; 2 Department of Microbiology, Sapthagiri Institute of Medical Sciences and Research Center, Bangalore, India; 3 Department of Ageing Research, Manipal School of Life Sciences, Manipal Academy of Higher Education 76793, Manipal, India; University of Pittsburgh School of Medicine, Pittsburgh, Pennsylvania, USA

**Keywords:** *Brucella melitensis*, clinical isolate, bloodstream infections

## Abstract

*Brucella melitensis* is the most virulent *Brucella* species causing human brucellosis. We report here the assembled and annotated genome sequence of *Brucella melitensis* isolates BBM21 and BVC23 obtained from two different patients in Karnataka, India. The genome size of the isolates are 3.31 Mb and 3.48 Mb, respectively.

## ANNOUNCEMENT

Brucellosis is an important zoonotic bacterial disease with significant public health impact globally. Among several pathogenic species, *Brucella melitensis* is the commonest and most virulent species causing human infections. Despite its huge implications on the economy and public health, the genomic data on *B. melitensis* clinical isolates from India are limited (1, 2). The whole-genome sequencing of clinical strains is important to improve the understanding of pathogen diversity, virulence potential, and regional epidemiology (3). In this announcement, we report the assembled and annotated sequences of two *B. melitensis* isolates, i.e., BBM21 and BVC23, recovered from human blood samples collected in Kasturba Hospital, Manipal, Karnataka, India. The institutional ethics committee has approved the study with IEC no.: 9/2022.

The blood culture of patients was performed in BacT/ALERT System (bioMérieux). After flagging positive, subculture was done on 5% sheep blood agar and incubated at 37°C with 5% CO_2_ for 24–48 h. Both the isolates were Gram-negative coccobacilli, oxidase, and urease positive. The species-level identification was done by AMOS-PCR, and both isolates were identified as *B. melitensis* (4). For the genomic DNA extraction, both isolates were sub-cultured on 5% sheep blood agar and incubated at 37°C with 5% CO_2_ for 24–48 h, and genomic DNA was extracted using the QIAmp DNA mini kit (Qiagen). The DNA concentrations were 32.4 µg/mL and 30.6 µg/mL for the BBM21 and BVC23 isolates, respectively, with a purity (A_260_/A_280_) of 1.66.

The library preparation of the DNA samples was done by using Illumina TruSeq Nano DNA Library Prep kit, and the libraries were analyzed on 4200 Tape Station system. The whole-genome sequencing was carried out using Illumina Novaseq6000 platform, generating 8,869,376 and 7,288,163 paired-end reads (2 × 150 bp) for BBM21 and BVC23 isolates, respectively. The adapters and low-quality bases are removed from the raw reads using Trimmomatic ver. 0.36 (5). The filtered raw reads were assembled *de novo* using SPAdes ver. 3.13.1 (6), and the assembly statistics are mentioned in Table 1.

**TABLE 1:** Assembly statistics of draft genomes

Assembly statistics	BBM21	BVC23
Genome coverage	512×	477×
Genome size	3.31 Mb	3.48 Mb
No. of contigs	35	40
Total length of contigs	3,290,702 bp	3,296,784 bp
N50 value	299,125 bp	250,962 bp
GC content	57%	56.5%

The average nucleotide identity (ANI) value of both BBM21 and BVC23 isolates is 99.87% with type strain *Brucella melitensis* 16M, confirming the isolates identity as *Brucella melitensis*. The genome annotation was performed using NCBI prokaryotic Genome Annotation pipeline (PGAP) (7). A total of 3,345 and 3,353 genes were predicted in BBM21 and BVC23 isolates. Out of these, 3,286 and 3,294 genes were identified as protein-coding sequences (CDS) in BBM21 and BVC23 isolates, respectively. In addition, BBM21 was predicted to contain 6 rRNA genes and 49 tRNA genes, whereas BVC23 has 5 rRNA genes and 50 tRNA genes. These data will be helpful for the comparative genomic and epidemiological analysis to understand the *B. melitensis* diversity.

## Data Availability

The genome accession numbers and SRA IDs are JBDLOF000000000 and SRR28425991 for BBM21 and JBDLOE000000000 and SRR28456461 for BVC23.
